# Neonatal, infant and under-five mortalities in Nigeria: An examination of trends and drivers (2003-2013)

**DOI:** 10.1371/journal.pone.0182990

**Published:** 2017-08-09

**Authors:** Oyewale Mayowa Morakinyo, Adeniyi Francis Fagbamigbe

**Affiliations:** 1 Department of Environmental Health Sciences, University of Ibadan, Ibadan, Nigeria; 2 Department of Epidemiology and Medical Statistics, University of Ibadan, Ibadan, Nigeria; Centre Hospitalier Universitaire Vaudois, FRANCE

## Abstract

Neonatal (NMR), infant (IMR) and under-five (U5M) mortality rates remain high in Nigeria. Evidence-based knowledge of trends and drivers of child mortality will aid proper interventions needed to combat the menace. Therefore, this study assessed the trends and drivers of NMR, IMR, and U5M over a decade in Nigeria. A nationally representative data from three consecutive Nigeria Demographic and Household Surveys (NDHS) was used. A total of 66,158 live births within the five years preceding the 2003 (6029), 2008 (28647) and 2013 (31482) NDHS were included in the analyses. NMR was computed using proportions while IMR and U5 were computed using life table techniques embedded in Stata version 12. Probit regression model and its associated marginal effects were used to identify the predisposing factors to NMR, IMR, and U5M. The NMR, IMR, and U5M per 1000 live births in 2003, 2008 and 2013 were 52, 41, 39; 100, 75, 69; and 201, 157, 128 respectively. The NMR, IMR, and U5M were consistently lower among children whose mothers were younger, living in rural areas and from richer households. Generally, the probability of neonate death in 2003, 2008 and 2013 were 0.049, 0.039 and 0.038 respectively, the probability of infant death was 0.093, 0.071 and 0.064 while the probability of under-five death was 0.140, 0.112 and 0.092 for the respective survey years. While adjusting for other variables, the likelihood of infant and under-five deaths was significantly reduced across the survey years. Maternal age, mothers’ education, place of residence, child’s sex, birth interval, weight at birth, skill of birth attendant, delivery by caesarean operation or not significantly influenced NMR, IMR, and U5M. The NMR, IMR, and U5M in Nigeria reduced over the studied period. Multi-sectoral interventions targeted towards the identified drivers should be instituted to improve child survival.

## Introduction

Child mortality has been at the hub of health discourse since time immemorial. Governments, health professionals, and policymakers have reserved an exclusive interest in reducing the prevalence of childhood deaths globally [1]. This interest has not only extended into the international scene, it has led to the development of sound interventions to reducing child mortality among children under the age of five between 1990 and 2015, and between 2015 and 2030 as tagged in the United Nation’s Millennium Development Goals (MDGs) [1], and Sustainable Development Goals (SDGs) respectively [2].

As recommended by different United Nations (UN) organisations, most countries use the reduction in under-five and maternal mortalities as bases for key development [3]. It’s also pertinent for countries to estimate their neonatal and infant mortalities so as develop appropriate intervention programs to reduce preventable child deaths. Neonatal mortality remains a significant public health problem worldwide. In the year 2015, 2.7 million deaths occurred in the first 28 days of life; representing a significant reduction to 19 deaths per 1,000 live births from the previously 36 deaths per 1000 live births in 1990 [4]. Though there was a decline in neonatal rates in some sub-Sahara African countries, such as Ghana and Uganda [5], the Nigerian Neonatal Mortality Rate (NMR) reduced by 20.4%, from 49 deaths per 1000 live births in 1990 to 39 in 2011 [6], 37 in 2013 [7] and to 34 deaths per 1000 live births in 2015 [8]. Globally, Nigeria ranks second to India with the highest number of neonatal deaths [6].

Similarly, Under-Five Mortality Rates (U5M) remain high in Nigeria despite recent interventions. A U5M of 128 deaths per 1000 live births as evidenced in the year 2013 Nigeria Demographic Health Survey suggests that about one in every eight children in Nigeria dies before their fifth birthday—approximately 21 times the average rate in developed countries [9]. Spanning a period of over two decades (1990 to 2015) in Nigeria, Infant Mortality Rates (IMR) have reduced by 57% (from 126 to 69 deaths per 1000 births); U5M fell by approximately 49% (from 213 to 109 deaths) [8].

Child deaths commonly result from several risk factors and preventable diseases. Acute Respiratory Infections (ARIs), diarrhoea, malaria and chronic malnutrition contribute largely to disease morbidity and mortality among children [10]. Deaths in the first 28 days of life has been linked to the endogenous (genetically-induced malfunctions, premature births) status of a child, quality of antenatal care, whether assistance was given during delivery and post-partum care [11]. However, deaths in the succeeding 11 months are often tied to the wealth status of the households, environmental factors, health behaviour and nutritional practices [11]. Several other factors have been linked with infant and child deaths. Such factors among others include maternal education, early marriage, place of residence, regional variations, short birth intervals, fertility behaviour, breastfeeding practices, use of health services by mother and or child, child’s sex, ethnicity and religion [12–16].

Notwithstanding the considerable gains achieved in reducing childhood deaths globally, the recorded progress was insufficient in meeting the MDG 4 target. The recommended SDG target for child mortality signifies a renewed commitment to the world’s children. Achieving this renewed commitment is dependent on monitoring the drivers of preventable deaths among children. The outcome of this study would serve as a bedrock for planning suitable interventions for halting preventable deaths of newborns and under-fives. Therefore, this study presents a population-based study on the trends and drivers of neonatal, infant and under-five mortalities over a decade in Nigeria.

## Methods

### Ethical consideration

The Institutional Review Board (IRB) of the National Institute of Medical Research, Nigeria approved the study protocol, survey instrument, and materials prior to the commencement of the surveys. Details of the ethical approvals have been reported earlier [17]. Informed consent was obtained from all parents and guardians who participated in the surveys.

### Study setting

Nigeria consists of 6 geopolitical regions; North-East, North-West, North-Central, South-East, South-South, and South-West which are sub-divided into 36 administrative states and the Federal Capital Territory (FCT). The population in each of the geopolitical regions and states are relatively homogeneous and share similar socio-cultural characteristics. Also, health-related characteristics like access to health care, environment, housing system etc. are similar within the regions and states.

### Sources of data and sampling

We pooled data from three consecutive nationally representative Nigeria Demographic and Household Surveys (NDHS) in 2003, 2008 and 2013. The survey uses three-stage sampling technique to select the respondents. Firstly, Local Government Areas (LGAs) are selected, then the Enumeration Areas (EA), which are the Primary Sampling Units (PSU) and referred to as clusters and lastly the selection of households within the selected EAs. Primary information about households, sexual and reproductive health and history were collected from women aged 15–49 years within the selected households. Usually, the survey collects birth history of all women interviewed. More specifically, the survey collects information on all births to a woman. We, therefore, used the “child recode data” which contains all follow-up information on all children born to the interviewed women within five years preceding the survey.

### Data analysis

Among the 7620, 33385 and 38948 women who participated in 2003, 2008, and 2013 surveys respectively, there were 6029, 28647 and 31482 children born within five years preceding each of the surveys. All analysis in this study were therefore based on the survivorship of the 66158 children within first five years of their life.

### Variables

There are three outcome variables in this study, they are neonatal deaths, infant deaths, and under-five children (U5) deaths. According to the NDHS, neonatal deaths, infant deaths, and under-five children (U5) deaths are deaths within the first 28 days, one year and five years respectively [18].

Based on past literature, the independent variables included in this study are:

1. Sociodemographic characteristics of mothers: maternal age (15–19, 20–24; 25–34; 35+), marital status (never married, currently married or living with sexual partner, formerly married), mother education (none, primary, secondary, higher), household wealth (poorest, middle, richest), religion (Islam, Christian/Catholic, others), media use (not at all, at least once a week, less than once a week), Residence (urban, rural), zone (North Central; North East; North West; South East; South-South; South West).
2. Characteristics of child: sex (male, female), birth order (1, 2–3, 4–6, 7+), birth interval (1st birth, <24 months, 24-47months, 48+ months), weight at birth (average or higher, small, very small), delivery mode (normal, caesarean).
3. Perceived benefit/need of health care: adequacy of antenatal care (ANC) use, based on the World Health Organisation (WHO) recommended four visits (none, inadequate, adequate), the skill of birth attendant (none, skilled, unskilled), tetanus injection received (yes, no).
4. Environmental characteristics: drinking water sources (unimproved, improved), toilet type (unimproved, improved), cooking fuel (unclean/biomass, clean fuel).

The groupings of the environmental characteristics were in tandem with those adopted in the 2013 NDHS [18] and the 2010 WHO and UNICEF document on progress on sanitation and drinking water [18]. The “source of drinking water” was grouped into either improved or not. Improved sources include piped into dwelling/yard/plot, public tap/standpipe, tube-well or borehole, protected well and spring, rain water, and bottle water. The improved toilet types are “flush/pour flush to piped sewer system”, “flush/pour flush to septic tank”, “flush/pour flush to pit latrine”, “ventilated improved pit (VIP) latrine”, “pit latrine with slab or composting toilet” while any other types of toilet facilities were categorised as non-improved.

### Statistical analysis

Descriptive statistics were used to show the distribution of the under-five children by the studied characteristics in Table 1. We then computed the NMR using proportions while IMR and U5M were computed using life table techniques embedded in Stata version 12 as presented in Table 2. Bivariate analyses were carried out to determine the significant association between each of the outcome variables and the independent variables using Pearson Chi-square (*x*^*2*^) test of association and also presented in Table 2. Probit regression model was used to identify the predisposing factors to neonatal death, infant mortality and under-five mortality.

**Table 1:** Distribution of live births followed up by years of DHS.

Characteristics	2003	2008	2013	Total	Total
	n = 6029	n = 28647	n = 31482	n = 66158	N = 66158
*Age*					
15–19	7.0	5.3	5.0	5.3	3509
20–24	21.1	19.5	19.6	19.7	13037
25–29	30.0	28.4	27.9	28.3	18728
30–39	32.8	36.4	37.4	36.6	24186
40–49	9.1	10.4	10.1	10.1	6698
*Marital Status*					
Never Married	2.0	1.7	1.6	1.6	1089
Living with sexual partner	95.0	95.7	95.8	95.7	63298
Widowed/Deceased/Separated	3.0	2.7	2.6	2.7	1770
*Wealth Status*					
Poorest	44.6	46.0	46.7	46.2	30550
Middle	20.2	19.3	18.9	19.2	12675
Richest	35.2	34.7	34.5	34.7	22933
*Education*					
No Education	51.8	46.5	49.2	48.3	31937
Primary	23.6	23.2	19.3	21.4	14130
Secondary	21.2	24.9	25.8	25.0	16531
Higher	3.5	5.4	5.8	5.4	3563
*Locality*					
Urban	28.9	29.8	35.0	32.1	21269
Rural	71.1	70.3	65.0	67.9	44889
*Zone*					
North Central	14.4	13.6	13.6	13.7	9065
North East	23.7	16.3	17.5	17.5	11610
North West	34.8	31.2	37.0	34.3	22690
South East	6.0	9.7	8.9	9.0	5953
South South	12.7	13.1	9.2	11.2	7406
South West	8.5	16.1	13.7	14.3	9432
*Religion*					
Islam	14.5	55.3	61.9	54.7	36179
Other Christian	11.6	33.7	28.1	29.0	19192
Catholics	9.3	9.3	8.5	8.9	5914
Others	64.6	1.7	1.6	7.4	4873
*Media*					
Not At All	27.3	27.3	37.4	32.1	21248
At least once a week	58.8	49.3	40.6	46.1	30467
Less than once a week	13.9	13.2	22.0	17.5	11550
*Child Sex*					
Male	51.2	50.9	50.5	50.7	33538
Female	48.8	49.2	49.6	49.3	32620
*Birth Interval*					
1st Birth	20.6	19.1	19.5	19.5	12875
<24 Months	19.2	19.2	18.7	19.0	12555
24-47months	46.6	47.4	47.4	47.3	31294
48+ Months	13.6	14.3	14.4	14.3	9437
*Weight at Birth*					
Average or higher	85.3	85.5	84.8	85.1	56331
Small	8.2	9.8	10.7	10.1	6650
Very Small	6.5	4.7	4.5	4.8	3176
*Tetanus received*					
No	48.2	44.9	40.5	43.1	28501
Yes	51.8	55.1	59.6	56.9	37657
*Number of antenatal visits*					
None	37.7	40.0	35.1	37.5	24779
Below 4	13.9	10.6	12.4	11.8	7789
4+	48.4	49.4	52.5	50.8	33590
*Skill of birth attendant*					
None	17.8	20.7	14.8	17.6	11645
Skilled	33.2	38.5	37.4	37.5	24803
Unskilled	49.0	40.8	47.9	44.9	29713
*Delivery mode*					
Normal	98.3	98.2	97.9	98.1	64883
Caesarean	1.8	1.8	2.1	1.9	1275
*Source of drinking water*					
Unimproved sources	28.0	47.7	40.2	42.3	27984
Improved sources	72.0	52.4	59.8	57.7	38174
*Toilet type*					
Unimproved sources	24.8	48.4	49.6	46.8	30965
Improved sources	75.2	51.7	50.4	53.2	35193
*Cooking fuel*					
Unclean/Biomass	80.8	99.0	98.5	97.1	64219
Clean fuel	19.2	1.0	1.6	2.9	1939
Total	9.1	43.3	47.6	100.0	

**Table 2:** Estimated neonatal, infant and under 5 mortalities per 1000 live births by selected children characteristics and DHS year.

Characteristics	NMR			IMR			U5M		
	2003	2008	2013	2003	2008	2013	2003	2008	2013
*Age*									
15–19	81	70	68	145	117	116	423	180	219
20–24	53	43	43	103	88	74	204	185	132
25–29	42	33	35	94	69	65	185	149	113
30–39	52	38	36	101	75	67	189	156	116
40–49	56	55	53	118	89	83	244	177	139
*Marital status*									
Never married	16	34	59	77	68	97	172	120	116
Living with sexual partner	52	41	40	104	79	74	200	163	124
Widowed/Deceased/Separated	59	55	58	112	97	92	275	190	147
*Wealth Status*									
Poorest	62	45	46	136	89	86	252	195	167
Middle	58	36	39	99	80	66	202	164	106
Richest	33	37	33	64	63	56	137	110	76
*Education*									
No Education	57	42	43	118	86	80	244	192	156
Primary	53	45	48	111	80	75	203	155	119
Secondary	38	37	35	67	71	59	120	125	83
Higher	49	30	21	67	43	45	92	61	55
*Locality*									
Urban	40	33	33	77	64	56	153	121	82
Rural	58	44	44	118	85	79	230	179	145
*Zone*									
North Central	45	39	37	97	77	61	163	150	95
North East	63	43	40	114	86	74	245	188	142
North West	51	41	48	110	80	84	241	196	160
South East	46	44	38	100	93	82	157	147	116
South South	53	47	31	110	80	55	173	136	85
South West	37	33	39	68	57	57	116	93	77
*Religion*									
Islam	54	40	42	93	79	75	164	178	144
Other Christian	57	42	38	94	76	65	164	136	96
Catholics	36	50	39	101	85	74	137	152	96
Others	53	50	50	108	94	69	232	167	109
*Media*									
Not at all	58	40	47	120	84	84	223	193	157
At least once a week	52	40	34	96	76	58	197	140	96
Less than once a week	38	49	41	105	83	77	188	177	124
*Child Sex*									
Male	61	50	46	110	87	78	199	170	134
Female	42	33	36	97	72	65	206	156	114
*Birth Interval*									
1st Birth	65	47	48	118	86	80	209	160	125
<24 Months	73	64	57	141	124	101	249	238	175
24-47months	42	35	33	91	69	60	202	148	112
48+ Months	36	24	29	71	45	52	126	109	85
*Birth Order*									
1	65	47	48	118	86	80	209	160	125
2	33	38	36	78	71	63	164	138	107
3	40	33	33	92	70	58	203	142	104
4	40	35	35	80	62	66	154	133	112
5	61	45	44	9	91	79	233	199	145
*Weight at Birth*									
Average or Higher	44	32	31	90	70	62	188	147	111
Small	55	48	56	110	94	94	213	185	156
Very Small	84	82	78	183	137	134	267	217	212
*Tetanus received*									
No	42	31	31	91	60	58	177	148	118
Yes	27	28	29	53	55	48	104	101	72
*Antenatal visits*									
None	44	31	33	91	61	64	197	153	122
Below 4	44	27	36	83	56	55	132	99	93
4+	24	27	27	52	53	46	97	101	73
*Skill of birth attendant*									
None	56	39	44	115	79	77	260	179	161
Skilled	40	43	39	71	73	61	134	116	85
Unskilled	59	41	40	114	78	73	218	175	135
*Delivery Mode*									
Normal	52	41	40	104	79	71	206	164	125
Ceaserian	48	81	62	105	120	96	118	155	109
*Source of drinking water*									
Unimproved sources	55	45	43	119	88	78	206	183	142
Improved sources	49	37	39	96	70	66	198	143	112
*Toilet Type*									
Unimproved sources	63	41	44	134	83	78	226	169	138
Improved sources	46	41	37	91	75	64	190	157	109
*Cooking Fuel*									
Unclean/Biomass	52	41	41	110	80	72	219	164	125
Clean Fuel	41	48	38	71	45	47	114	66	55
Total	52	41	41	104	79	72	203	164	124

In probit regression model, attempt is made to model the (conditional) probability of a "successful" outcome, that is,

$$\mathbb{P}\left(\Upsilon_{l}\;=\;1|{Χ}_{1l},\;\ldots ,\;{Χ}_{\kappa l};\;\beta_{0},\;\ldots ,\;\beta_{\kappa }\right)=\Phi (\beta_{0}\;+\;\sum_{\kappa =1}^{Κ}\beta_{\kappa }{Χ}_{\kappa l})$$ (1)

It is expressed as a derivative of Eq (1) as shown in Eq (2)

$$\frac{\left(\partial \mathbb{P}\left(\Upsilon_{\_l}\;=\;1{|Χ}_{\_\left(1l\right)},\;\ldots ,\;{Χ}_{\_\kappa l};\;\beta_{\_0},\;\ldots ,\;\beta_{\_\kappa }\right)\right)}{\left(\partial {Χ}_{\_\kappa l}\right)}\;=\;\beta_{\_\kappa }\Phi \left(\beta_{\_0}\;+\;\sum_{\kappa \;=1}^{Κ}\left(\beta_{\_k}{Χ}_{\_\kappa l}\right)\right)$$ (2)

where Φ(·) is the cumulative distribution function of the standard normal distribution. That is, conditional on the explanatory variables, the probability that the outcome variable, Yi = 1, is a certain function of a linear combination of the explanatory variables. A positive regression coefficient indicates that an increase in the predictor leads to an increase in the predicted probability while a negative coefficient is an indication that t an increase in the predictor would reduce the predicted probability.

We provided the marginal effects of the explanatory variables. The marginal effects estimated using the “delta method” involves the use of calculus to show how much the (conditional) probability of the outcome variable changes when there is a change in the value of an explanatory variable, holding all other explanatory constant at their values. It is worth noting that unlike the linear regression case where the estimated regression coefficients are the marginal effects, there is a need for the additional level of computation to estimate the marginal effects haven computed the probit regression.

In the case of a discrete explanatory variable, the change in the probability is

$$\begin{array}{l} \begin{array}{l} {\Delta \chi }_{\kappa \tau }\mathbb{P}\left(\Upsilon_{i}=1{|Χ}_{1i},\;\ldots ,\;{Χ}_{\kappa i};\;\beta_{0},\;\ldots ,\;\beta_{\kappa }\right)=\beta_{\kappa }\phi (\beta_{0}\;+\;\sum_{l\;=1}^{Κ-1}\beta_{l}{Χ}_{li}+\;\beta_{\kappa }\;+\\ \sum_{l\;=\kappa +1}^{Κ}\beta_{l}{Χ}_{li})\;-\;\beta_{\kappa }\phi (\beta_{0}\;+\;\sum_{l\;=1}^{Κ-1}\beta_{l}{Χ}_{li}\;+\;\beta_{\kappa }\;+\;\sum_{l\;=\kappa +1}^{Κ}\beta_{l}{Χ}_{li}) \end{array} \end{array}$$ (3)

Sampling weights were applied, statistical significance was determined at 5% and Stata 12 used for all analysis while multicollinear variables were removed in the final model. There are four distinct columns in the Tables 3, 4, 5 and 6. The first column is the marginal effects computed from the coefficients of the probit model. It shows changes in a particular category with respect to the reference category. The second column is the standard error of the estimate in the 1st column while the 3rd column is the associated p-value. However, the 4th column presented the estimated increase/reduction per 1000 live births with respect to the reference category.

**Table 3:** Factors influencing neonate deaths using probit regression model 2003–2013.

Characteristics	2003				2008				2013			
	Mar Eff	SE	Sig	MPT	Mar Eff	SE	Sig	MPT	Mar Eff	SE	Sig	MPT
*Age*												
15–19	Ref											
20–24	-0.022	0.014	0.127	-22	-0.020	0.007	0.002	-20	-0.018	0.007	0.006	-18
25–29	-0.031	0.014	0.023	-31	-0.029	0.006	0.000	-29	-0.027	0.006	0.000	-27
30–39	-0.017	0.014	0.215	-17	-0.025	0.006	0.000	-25	-0.025	0.006	0.000	-25
40–49	-0.014	0.016	0.392	-14	-0.009	0.007	0.242	-9	-0.012	0.007	0.091	-12
*Marital Status*												
Living With SP	Ref											
Never Married	-0.032	0.013	0.017	-32	-0.005	0.008	0.507	-5	0.012	0.009	0.169	12
Widowed/Deceased/Separated	-0.007	0.014	0.597	-7	0.008	0.008	0.287	8	0.012	0.007	0.099	12
*Wealth Status*												
Poorest	Ref											
Middle	-0.011	0.008	0.181	-11	-0.007	0.003	0.028	-7	-0.007	0.003	0.015	-7
Richest	-0.029	0.006	0.000	-29	-0.007	0.003	0.004	-7	-0.011	0.002	0.000	-11
*Education*												
No Education	Ref											
Primary	-0.010	0.007	0.138	-10	0.002	0.003	0.410	2	0.002	0.003	0.454	2
Secondary	-0.021	0.007	0.001	-21	-0.002	0.003	0.458	-2	-0.008	0.003	0.003	-8
Higher	-0.005	0.016	0.738	-5	-0.011	0.005	0.021	-11	-0.016	0.004	0.000	-16
*Locality*												
Urban	-0.017	0.006	0.003	-17	-0.007	0.002	0.003	-7	-0.011	0.002	0.000	-11
Rural	Ref											
*Zone*												
North Central	Ref											
North East	0.013	0.009	0.127	13	0.004	0.004	0.234	4	0.005	0.004	0.144	5
North West	0.010	0.008	0.207	10	0.000	0.003	0.998	0	0.008	0.003	0.017	8
South East	0.014	0.012	0.236	14	0.010	0.005	0.054	10	0.006	0.005	0.206	6
South South	0.012	0.011	0.284	12	0.009	0.004	0.049	9	-0.003	0.004	0.390	-3
South West	-0.009	0.009	0.328	-9	-0.005	0.004	0.196	-5	0.002	0.004	0.611	2
*Religion*												
Islam	Ref											
Other Christian	0.001	0.010	0.947	1	0.005	0.003	0.073	5	-0.003	0.002	0.247	-3
Catholics	-0.009	0.011	0.398	-9	0.013	0.005	0.005	13	-0.001	0.004	0.861	-1
Others	0.004	0.008	0.597	4	0.008	0.009	0.349	8	0.001	0.009	0.897	1
*Media exposure*												
Not at all	Ref											
At least once a week	-0.007	0.007	0.306	-7	0.001	0.002	0.670	1	-0.010	0.002		-10
Less than once a week	-0.008	0.009	0.394	-8	0.008	0.004	0.032	8	-0.003	0.003	0.290	-3
*Child Sex*												
Male	Ref											
Female	-0.019	0.006	0.001	-19	-0.011	0.002	0.000	-11	-0.010	0.002	0.000	-10
*Birth interval*												
1st Birth	Ref											
<24 months	0.004	0.010	0.679	4	0.014	0.004	0.001	14	0.008	0.004	0.062	8
24–47 months	-0.022	0.008	0.004	-22	-0.014	0.003	0.000	-14	-0.017	0.003	0.000	-17
48+ months	-0.026	0.009	0.006	-26	-0.025	0.004	0.000	-25	-0.018	0.004	0.000	-18
*Birth order*												
1	Ref											
2	-0.031	0.009	0.000	-31	-0.009	0.004	0.028	-9	-0.014	0.004	0.000	-14
3	-0.025	0.009	0.007	-25	-0.017	0.004	0.000	-17	-0.015	0.004	0.000	-15
4	-0.027	0.010	0.005	-27	-0.017	0.004	0.000	-17	-0.013	0.004	0.001	-13
5	-0.003	0.009	0.713	-3	-0.003	0.004	0.394	-3	-0.007	0.003	0.034	-7
*Weight at birth*												
Average or higher	-0.040	0.014	0.005	-40	-0.050	0.007	0.000	-50	-0.047	0.007	0.000	-47
Small	-0.029	0.018	0.100	-29	-0.034	0.008	0.000	-34	-0.022	0.008	0.008	-22
Very small	Ref											
*Tetanus received*												
No	Ref											
Yes	-0.013	0.006	0.028	-13	0.001	0.002	0.736	1	-0.003	0.002	0.267	-3
Antenatal visits												
None	Ref											
Below 4	-0.001	0.010	0.958	-1	-0.002	0.004	0.572	-2	0.000	0.004	0.953	0
4+	-0.015	0.006	0.018	-15	-0.001	0.003	0.831	-1	-0.007	0.003	0.008	-7
*Skill of birth attendant*												
None	Ref											
Skilled	-0.018	0.008	0.035	-18	0.006	0.003	0.054	6	-0.001	0.004	0.688	-1
Unskilled	0.001	0.008	0.883	1	0.002	0.003	0.543	2	-0.001	0.003	0.845	-1
*Delivery Mode*												
Normal	-0.009	0.023	0.696	-9	-0.052	0.013	0.000	-52	-0.036	0.010	0.000	-36
Caesarean	Ref											
*Drinking water source*												
Unimproved	Ref											
Improved Source	-0.007	0.006	0.295	-7	-0.006	0.002	0.007	-6	-0.003	0.002	0.195	-3
*Toilet Type*												
Unimproved	Ref											
Improved	-0.018	0.007	0.008	-18	-0.002	0.002	0.443	-2	-0.006	0.002	0.006	-6
*Cooking fuel*												
Unclean/Biomass	Ref											
Clean Fuel	-0.014	0.077	0.069	-14	-0.003	0.012	0.803	-3	-0.003	0.008	0.759	-3
Total	0.049	0.003		49	0.039	0.001		39	0.038	0.001		38

Mar Eff Marginal Effect, SE Standard Error Sig. Significance MPT Mortality per 1000 Livebirths to Differences in Mortality per 1000 Livebirths

**Table 4:** Factors affecting infant mortality from probit regression model 2003–2013.

Characteristics	2003				2008				2013			
	Mar Eff	SE	Sig	MPT	Mar Eff	SE	Sig	MPT	Mar Eff	SE	Sig	MPT
*Age*												
15–19	Ref											
20–24	-0.025	0.018	0.165	-25	-0.019	0.008	0.023	-19	-0.030	0.008	0.000	-30
25–29	-0.032	0.018	0.067	-32	-0.034	0.008	0.000	-34	-0.037	0.008	0.000	-37
30–39	-0.026	0.018	0.139	-26	-0.028	0.008	0.000	-28	-0.034	0.008	0.000	-34
40–49	-0.006	0.021	0.775	-6	-0.013	0.009	0.139	-13	-0.017	0.009	0.053	-17
*Marital status*												
Living with sexual partner	Ref											
Never Married	-0.029	0.024	0.229	-29	-0.014	0.010	0.191	-14	0.019	0.011	0.086	19
Widowed/Deceased/Separated	0.011	0.021	0.608	11	0.021	0.011	0.048	21	0.023	0.010	0.014	23
*Wealth Status*												
Poorest	Ref											
Middle	-0.032	0.010	0.002	-32	-0.008	0.004	0.056	-8	-0.019	0.004	0.000	-19
Richest	-0.065	0.008	0.000	-65	-0.023	0.003	0.000	-23	-0.028	0.003	0.000	-28
*Education*												
No education	Ref											
Primary	-0.006	0.010	0.529	-6	-0.005	0.004	0.177	-5	-0.005	0.004	0.171	-5
Secondary	-0.046	0.009	0.000	-46	-0.015	0.004	0.000	-15	-0.020	0.003	0.000	-20
Higher	-0.045	0.017	0.008	-45	-0.039	0.006	0.000	-39	-0.032	0.005	0.000	-32
*Locality*												
Urban	-0.036	0.007	0.000	-36	-0.019	0.003	0.000	-19	-0.021	0.003	0.000	-21
Rural	Ref											
*Zone*												
North Central	Ref											
North East	0.016	0.012	0.189	16	0.008	0.005	0.081	8	0.012	0.005	0.011	12
North West	0.012	0.011	0.281	12	0.004	0.005	0.445	4	0.020	0.004	0.000	20
South East	0.003	0.015	0.845	3	0.014	0.007	0.031	14	0.018	0.006	0.002	18
South South	0.012	0.015	0.454	12	0.003	0.006	0.653	3	-0.005	0.005	0.268	-5
South West	-0.027	0.013	0.036	-27	-0.018	0.005	0.000	-18	-0.004	0.005	0.406	-4
*Religion*												
Islam	Ref											
Other Christian	0.001	0.014	0.944	1	-0.003	0.003	0.355	-3	-0.009	0.003	0.004	-9
Catholics	0.007	0.015	0.648	7	0.005	0.006	0.400	5	-0.001	0.005	0.887	-1
Others	0.013	0.011	0.231	13	0.014	0.012	0.236	14	-0.004	0.011	0.724	-4
*Media exposure*												
Not at all	Ref											
At least once a week	-0.021	0.009	0.019	-21	-0.007	0.003	0.026	-7	-0.024	0.003	0.000	-24
Less than once a week	-0.013	0.013	0.297	-13	-0.001	0.005	0.787	-1	-0.007	0.004	0.072	-7
*Child Sex*												
Male	Ref											
Female	-0.012	0.007	0.108	-12	-0.013	0.003	0.000	-13	-0.012	0.003	0.000	-12
*Birth interval*												
1st Birth	Ref											
<24 months	0.025	0.013	0.063	25	0.038	0.006	0.000	38	0.022	0.005	0.000	22
24–47 months	-0.025	0.010	0.014	-25	-0.016	0.004	0.000	-16	-0.018	0.004	0.000	-18
48+ months	-0.044	0.012	0.000	-44	-0.037	0.005	0.000	-37	-0.026	0.005	0.000	-26
*Birth order*												
1	Ref											
2	-0.035	0.012	0.003	-35	-0.013	0.005	0.011	-13	-0.015	0.005	0.001	-15
3	-0.024	0.013	0.066	-24	-0.014	0.005	0.006	-14	-0.020	0.005	0.000	-20
4	-0.034	0.013	0.008	-34	-0.021	0.005	0.000	-21	-0.012	0.005	0.016	-12
5	0.001	0.011	0.924	1	0.004	0.005	0.332	4	-0.001	0.004	0.818	-1
*Weight at Birth*												
Average or higher	-0.085	0.019	0.000	-85	-0.057	0.009	0.000	-57	-0.064	0.009	0.000	-64
Small	-0.069	0.023	0.003	-69	-0.036	0.010	0.000	-36	-0.035	0.010	0.001	-35
Very small	Ref											
*Tetanus received*												
No	Ref											
Yes	-0.032	0.008	0.000	-32	-0.005	0.003	0.140	-5	-0.009	0.003	0.004	-9
*Antenatal visits*												
None	Ref											
Below 4	-0.009	0.013	0.479	-9	-0.006	0.005	0.263	-6	-0.009	0.005	0.061	-9
4+	-0.033	0.009	0.000	-33	-0.008	0.004	0.033	-8	-0.016	0.003	0.000	-16
*Birth attendant skill*												
None	Ref											
Skilled	-0.040	0.011	0.000	-40	-0.006	0.004	0.176	-6	-0.015	0.005	0.001	-15
Unskilled	-0.002	0.011	0.882	-2	0.000	0.004	0.956	0	-0.004	0.005	0.367	-4
*Delivery mode*												
Normal	-0.004	0.029	0.886	-4	-0.037	0.014	0.010	-37	-0.021	0.011	0.054	-21
Caesarean	Ref											
*Source of drinking water*												
Unimproved	Ref											
Improved source	-0.021	0.009	0.014	-21	-0.017	0.003	0.000	-17	-0.011	0.003	0.000	-11
*Toilet Type*												
Unimproved	Ref											
Improved	-0.038	0.009	0.000	-38	-0.007	0.003	0.033	-7	-0.013	0.003	0.000	-13
*Cooking fuel*												
Unclean/Biomass	Ref											
Clean Fuel	-0.036	0.009	0.000	-36	-0.032	0.012	0.011	-32	-0.021	0.009	0.019	-21
Total	0.093	0.004		93	0.071	0.002		71	0.064	0.001		64

Mar Eff Marginal Effect, SE Standard Error Sig. Significance MPT Mortality per 1000Livebirthto Differences in Mortality per 1000 Livebirths

**Table 5:** Factors affecting under-five mortality from probit regression model 2003–2013.

Characteristics	2003				2008				2013			
	Mar Eff	SE	Sig	MPT	Mar Eff	SE	Sig	MPT	Mar Eff	SE	Sig	MPT
*Age*												
15–19	Ref											
20–24	-0.013	0.021	0.538	-13	0.000	0.009	0.975	0	-0.026	0.009	0.004	-26
25–29	-0.027	0.020	0.183	-27	-0.018	0.009	0.041	-18	-0.036	0.009	0.000	-36
30–39	-0.016	0.020	0.408	-16	-0.010	0.009	0.263	-10	-0.031	0.009	0.000	-31
40–49	0.028	0.024	0.255	28	0.015	0.010	0.147	15	-0.007	0.010	0.477	-7
*Marital status*												
Living with sexual partner	Ref											
Never Married	-0.048	0.028	0.090	-48	-0.028	0.012	0.024	-28	-0.001	0.012	0.899	-1
Widowed/Deceased/Separated	0.040	0.027	0.135	40	0.045	0.013	0.001	45	0.028	0.011	0.010	28
*Wealth Status*												
Poorest	Ref											
Middle	-0.038	0.013	0.003	-38	-0.016	0.005	0.001	-16	-0.039	0.004	0.000	-39
Richest	-0.092	0.010	0.000	-92	-0.050	0.004	0.000	-50	-0.057	0.004	0.000	-57
*Education*												
No education	Ref											
Primary	-0.031	0.011	0.006	-31	-0.019	0.005	0.000	-19	-0.020	0.004	0.000	-20
Secondary	-0.090	0.010	0.000	-90	-0.042	0.005	0.000	-42	-0.047	0.004	0.000	-47
Higher	-0.091	0.020	0.000	-91	-0.085	0.006	0.000	-85	-0.068	0.005	0.000	-68
*Locality*												
Urban	-0.058	0.009	0.000	-58	-0.039	0.004	0.000	-39	-0.041	0.003	0.000	-41
Rural	Ref											
*Zone*												
North central	Ref											
North east	0.049	0.014	0.001	49	0.025	0.006	0.000	25	0.030	0.005	0.000	30
North west	0.042	0.013	0.002	42	0.032	0.006	0.000	32	0.045	0.005	0.000	45
South east	-0.008	0.017	0.658	-8	0.013	0.008	0.086	13	0.022	0.007	0.001	22
South south	0.017	0.018	0.322	17	-0.001	0.007	0.917	-1	-0.005	0.006	0.404	-5
South west	-0.039	0.015	0.008	-39	-0.034	0.006	0.000	-34	-0.010	0.005	0.078	-10
*Religion*												
Islam	Ref											
Other Christian	-0.006	0.016	0.689	-6	-0.025	0.004	0.000	-25	-0.028	0.003	0.000	-28
Catholics	-0.006	0.017	0.717	-6	-0.016	0.007	0.021	-16	-0.024	0.006	0.000	-24
Others	0.037	0.013	0.003	37	-0.007	0.013	0.610	-7	-0.014	0.013	0.284	-14
*Media exposure*												
Not at all	Ref											
At least once a week	-0.037	0.011	0.001	-37	-0.026	0.004	0.000	-26	-0.041	0.004	0.000	-41
Less than once a week	-0.036	0.015	0.015	-36	-0.005	0.006	0.427	-5	-0.018	0.005	0.000	-18
*Child sex*												
Male	Ref											
Female	-0.008	0.009	0.378	-8	-0.014	0.004	0.000	-14	-0.015	0.003	0.000	-15
*Birth interval*												
1st birth	Ref											
<24 months	0.046	0.016	0.004	46	0.067	0.007	0.000	67	0.043	0.006	0.000	43
24–47 months	-0.020	0.012	0.098	-20	-0.009	0.005	0.061	-9	-0.015	0.004	0.001	-15
48+ months	-0.061	0.014	0.000	-61	-0.039	0.006	0.000	-39	-0.034	0.005	0.000	-34
*Birth order*												
1	Ref											
2	-0.041	0.014	0.004	-41	-0.010	0.006	0.100	-10	-0.015	0.005	0.004	-15
3	-0.021	0.015	0.175	-21	-0.012	0.006	0.062	-12	-0.018	0.005	0.001	-18
4	-0.037	0.016	0.017	-37	-0.017	0.006	0.008	-17	-0.010	0.006	0.071	-10
5	0.013	0.013	0.300	13	0.023	0.005	0.000	23	0.009	0.005	0.057	9
*Weight at birth*												
Average or higher	-0.090	0.022	0.000	-90	-0.060	0.010	0.000	-60	-0.074	0.010	0.000	-74
Small	-0.066	0.026	0.012	-66	-0.030	0.012	0.010	-30	-0.039	0.011	0.001	-39
Very small	Ref											
*Tetanus received*												
No	Ref											
Yes	-0.043	0.009	0.000	-43	-0.020	0.004	0.000	-20	-0.018	0.003	0.000	-18
*Antenatal visits*												
None	Ref											
Below 4	-0.034	0.015	0.023	-34	-0.020	0.006	0.001	-20	-0.016	0.006	0.004	-16
4+	-0.055	0.011	0.000	-55	-0.025	0.004	0.000	-25	-0.025	0.004	0.000	-25
*Skill of birth attendant*												
None	Ref											
Skilled	-0.091	0.014	0.000	-91	-0.036	0.005	0.000	-36	-0.046	0.006	0.000	-46
Unskilled	-0.029	0.014	0.040	-29	-0.005	0.005	0.315	-5	-0.015	0.006	0.009	-15
*Delivery mode*												
Normal	0.034	0.031	0.266	34	-0.018	0.016	0.254	-18	-0.001	0.011	0.937	-1
Caesarean	Ref											
*Source of drinking water*												
Unimproved	Ref											
Improved source	-0.015	0.010	0.133	-15	-0.028	0.004	0.000	-28	-0.021	0.003	0.000	-21
*Toilet type*												
Unimproved	Ref											
Improved	-0.034	0.010	0.001	-34	-0.007	0.004	0.045	-7	-0.020	0.003	0.000	-20
Cooking fuel												
Unclean/Biomass	Ref											
Clean Fuel	-0.068	0.010	0.000	-68	-0.064	0.014	0.000	-64	-0.043	0.010	0.000	-43
Total	0.140	0.004		140	0.112	0.002		112	0.092	0.002		92

Mar Eff Marginal Effect, SE Standard Error Sig. Significance MPT Mortality per 1000 Livebirths to Differences in Mortality per 1000 Livebirths

**Table 6:** Adjusted risk factors of neonatal, infant and under-five mortality in Nigeria from probit regression model 2003–2013.

Characteristics	Neonatal Mortality				Infant Mortality				Under-five mortality			
	Mar Eff	SE	Sig	MPT	Mar Eff	SE	Sig	MPT	Mar Eff	SE	Sig	MPT
DHS (2003)												
2008	-0.004	0.004	0.351	-4	-0.016	0.006	0.011	-16	-0.023	0.007	0.002	-23
2013	-0.003	0.004	0.454	-3	-0.019	0.006	0.003	-19	-0.031	0.007	0.000	-31
Age (15–19)												
20–24	-0.012	0.004	0.003	-12	-0.015	0.005	0.004	-15	-0.007	0.005	0.212	-7
25–29	-0.016	0.004	0.000	-16	-0.019	0.005	0.000	-19	-0.009	0.006	0.100	-9
30–39	-0.010	0.005	0.021	-10	-0.013	0.006	0.025	-13	-0.001	0.006	0.915	-1
40–49	0.001	0.005	0.799	1	0.007	0.007	0.321	7	0.032	0.007	0.000	32
Marital Status (Currently Married)												
Never Married	-0.003	0.005	0.544	-3	0.003	0.007	0.689	3	0.009	0.009	0.297	9
Formerly Married	0.001	0.005	0.853	1	0.014	0.007	0.043	14	0.038	0.009	0.000	38
Wealth (Poorest)												
Middle	-0.002	0.002	0.448	-2	-0.003	0.003	0.416	-3	-0.003	0.004	0.428	-3
Richest	-0.001	0.003	0.867	-1	-0.005	0.004	0.196	-5	-0.006	0.005	0.188	-6
Education (None)												
Primary	0.002	0.003	0.523	2	0.001	0.003	0.871	1	0.001	0.004	0.740	1
Secondary	-0.004	0.003	0.163	-4	-0.007	0.004	0.068	-7	-0.010	0.004	0.025	-10
Higher	-0.008	0.004	0.040	-8	-0.013	0.006	0.020	-13	-0.023	0.006	0.000	-23
Residence (Urban)	-0.007	0.002	0.002	-7	-0.009	0.003	0.002	-9	-0.014	0.003	0.000	-14
Zone (North Central)												
North East	0.005	0.003	0.105	5	0.004	0.004	0.241	4	0.010	0.004	0.026	10
North West	0.002	0.003	0.413	2	0.006	0.004	0.145	6	0.014	0.004	0.002	14
South East	0.005	0.004	0.197	5	0.009	0.005	0.073	9	0.013	0.006	0.025	13
South South	0.004	0.003	0.214	4	0.003	0.004	0.468	3	0.004	0.005	0.430	4
South West	-0.002	0.003	0.422	-2	-0.004	0.004	0.348	-4	-0.006	0.005	0.234	-6
*Religion* (Islam)												
Other Christian	0.006	0.003	0.028	6	0.010	0.004	0.006	10	0.006	0.004	0.140	6
Catholics	0.002	0.004	0.672	2	0.004	0.005	0.356	4	0.001	0.006	0.857	1
Others	0.000	0.004	0.956	0	-0.003	0.005	0.594	-3	-0.004	0.006	0.452	-4
Media (None)												
At least once a week	0.001	0.002	0.794	1	-0.001	0.003	0.789	-1	0.002	0.003	0.526	2
Less than once a week	0.005	0.003	0.063	5	0.001	0.003	0.671	1	0.002	0.004	0.580	2
Sex (Female)	-0.010	0.002	0.000	-10	-0.010	0.002	0.000	-10	-0.013	0.002	0.000	-13
Birth interval (First)												
<24 months	0.004	0.004	0.273	4	0.013	0.005	0.006	13	0.017	0.006	0.002	17
24–47 months	-0.009	0.003	0.004	-9	-0.010	0.004	0.010	-10	-0.012	0.005	0.011	-12
48+ months	-0.014	0.003	0.000	-14	-0.021	0.004	0.000	-21	-0.028	0.005	0.000	-28
Weight at birth (Average/higher)												
Small	-0.033	0.005	0.000	-33	-0.036	0.006	0.000	-36	-0.035	0.007	0.000	-35
Very small	-0.024	0.006	0.000	-24	-0.024	0.007	0.001	-24	-0.023	0.008	0.002	-23
Had no tetanus Injection	0.001	0.003	0.794	1	0.003	0.004	0.484	3	0.005	0.004	0.257	5
Antenatal visit (None)												
1 to 3	0.001	0.003	0.752	1	-0.001	0.004	0.788	-1	-0.006	0.005	0.239	-6
4 or more	-0.002	0.003	0.496	-2	-0.003	0.004	0.510	-3	-0.006	0.005	0.231	-6
Skill of birth attendant (None)												
Skilled	0.009	0.003	0.004	9	0.009	0.004	0.036	9	0.005	0.005	0.319	5
Unskilled	0.003	0.002	0.145	3	0.003	0.003	0.349	3	0.002	0.004	0.476	2
Delivery mode (Caesarean)	0.042	0.009	0.000	42	0.042	0.011	0.000	42	0.047	0.012	0.000	47
Drinking water (Improved)	0.002	0.002	0.406	2	-0.002	0.002	0.504	-2	0.000	0.003	0.927	0
Toilet Type (Improved)	-0.002	0.002	0.303	-2	-0.003	0.002	0.306	-3	-0.003	0.003	0.224	-3
Cooking fuel (Clean)	0.005	0.006	0.405	5	-0.001	0.007	0.943	-1	0.003	0.009	0.769	3

Mar Eff Marginal Effect, SE Standard Error Sig. Significance MPT Mortality per 1000 Live births to Differences in Mortality per 1000 Livebirths

## Results

Over a third, (36.6%) of the mothers of the children interviewed were aged 30–39 years while 28.3% were aged 25–29 years. The majority (95.7%) were either currently married or living with sexual partners and nearly half, 46.2% are from households in the poorest wealth quintile. Regarding education, 48.3% had no formal education, 67.9% resided in a rural area while the sex ratio of the children was about 1 to 1 (Table 1).

### Multivariate analysis

**Fig 1:**
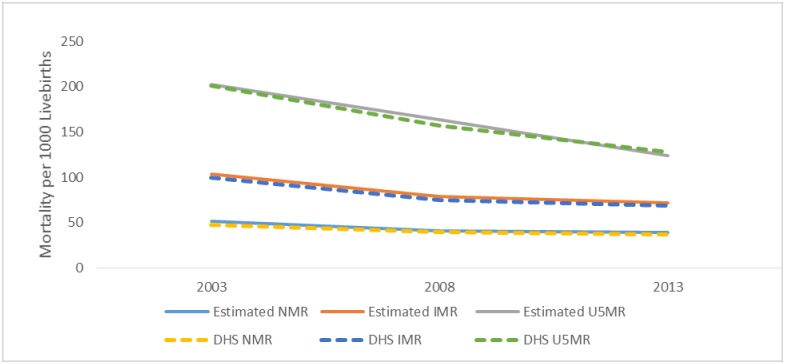
Trends of neonatal, infant and under 5 mortalities in Nigeria.

Each of the three indicators (NMR, IMR, and U5M) were analysed separately for each of the years as reported in Table 2. The NMR, IMR, and U5M seemed to have reduced in subsequent survey years. For instance, NMR fell from 52 per 1000 live births in 2003 through 41 in 2008 to 39 in 2013 (Table 2 and Fig 1). Similarly, the overall IMR (100, 75 and 69) and U5M (201, 157 and 128) reduced consistently in year 2003, 2008 and 2013 respectively. The NMR, IMR, and U5M were consistently lower among children whose mothers were younger than among those with older parents and also among the currently or formerly marrieds than the single mothers.

The mortality rates considered were also higher in rural areas than the urban areas and also higher among children from poorer households as shown in Table 2. On sex of child, NMR (61, 50 and 46), IMR (110, 87 and 84) and U5M (199, 170 and 134) were higher among males than the females at NMR (42, 33 and 36), IMR (97, 72 and 65) and U5M (206, 156 and 114) in 2003, 2008 and 2013 respectively. Similar patterns were noticed along the birth intervals and weights at the birth of the children as children with shorter birth intervals experienced higher mortalities and higher among children with smaller birth weights. Also, mortalities were significantly higher among children who had water from unimproved sources and used unimproved toilet types.

### Neonatal deaths

Generally, the probability of neonate deaths in 2003, 2008 and 2013 were 0.049, 0.039 and 0.038 respectively. Table 3 shows the outcomes of probit regression model used to identify significant determinants of neonatal deaths for each of the survey years. For instance, the probability of neonate deaths was 0.022 lower among children whose mothers were aged 20–24 years than those aged 15–19 years in 2003. Considering mother’ education, having higher education reduced the probability of neonate deaths by 0.016 in 2013. On the area of residence, living in an urban area reduced the chances of neonate deaths by 0.017 in 2003, 0.007 in 2008 and 0.011 in 2013. Similar trends were noticeable along the sources of drinking water and type of toilets used. Childs sex, Mothers’ age, education, wealth status, place and zone of residence, birth order and birth interval, adequacy of ANC visits and type of toilet facility were significant predictors of neonatal mortality in all the survey years. Marital status, religion, receiving tetanus injection after birth, skill of birth attendant were significant to neonate deaths in 2003 but was not significant in 2008 and 2013. However, delivery mode, media use was significant in 2008 and 2013 only, while the type of cooking fuel and source of drinking water was only significant in 2008.

### Infant death

The probability of infant death in 2003, 2008 and 2013 were 0.093, 0.071 and 0.064 respectively. In Table 4, we present the outcomes of probit regression model used to identify significant determinants of infant deaths for the respective survey years. The probability of infant death was 0.025, 0.019 and 0.030 lower among children whose mothers were aged 20–24 years than those aged 15–19 years in 2003, 2008 and 2013 respectively. Households in the richest wealth quintiles recorded a reduction in the number of infant deaths by 0.065, 0.023 and 0.028 in 2003, 2008 and 2013 respectively compared with children from poorest households. Considering mother’ education, compared with mothers with no education, having higher education reduced the probability of infant deaths by 0.045, 0.039 and 0.032 in 2003, 2008 and 2013 respectively. Similar trends were noticeable along the birth intervals, weight at birth, sources of drinking water and type of toilets used. Mothers’ education, wealth status, place and zone of residence, media use, birth order and birth interval, weight at birth, receiving tetanus injection after birth, adequacy of ANC visits, source of drinking water, type of cooking fuel and type of toilet facility were significant predictors of infant mortality in all the survey years. However, mothers’ age, marital status, child’s sex, delivery mode, were significant in 2008 and 2013 only.

### Under-five deaths

Overall, the probability of under-five death in 2003, 2008 and 2009 were 0.140, 0.112 and 0.092 respectively. In Table 5, the outcomes of probit regression model used to identify significant determinants of under-five deaths for the respective survey years are presented. The probability of under-five death was 0.013, 0.000 and 0.026 lower among children whose mothers were aged 20–24 years than those aged 15–19 years in 2003, 2008 and 2013 respectively. Been from households in richest in richest wealth quintiles reduced the chances of under-five death by 0.092, 0.050 and 0.057 in 2003, 2008 and 2013 respectively compared with children from poorest households. Considering mother’ education, compared with mothers with no education, having higher education reduced the probability of under-five deaths by 0.091, 0.085 and 0.068 in 2003, 2008 and 2013 respectively. Similar trends were noticeable in religion, sex of the child, birth intervals, weight at birth, sources of drinking water and type of toilets used. Mothers’ education, wealth status, place and zone of residence, religion, media use, birth order and birth interval, weight at birth, receiving tetanus injection after birth, adequacy of ANC visits, skill of birth attendants, type of cooking fuel and type of toilet facility were significant predictors of under-five mortality in all the survey years. Whereas, mothers’ age, marital status, child’s sex, the source of drinking water, were significant in 2008 and 2013 only.

The adjusted factors influencing neonatal, infant and under-five mortalities are presented in Table 6. In the presence of other variables, the probability of neonate death did not reduce significantly across the survey years, although the probability of infant and under-five deaths reduced significantly in the same period. While controlling for other variables, maternal age, mothers’ education, place of residence, religion, child’s sex, birth interval, weight at birth, skill of birth attendant, delivery by caesarean operation or not significantly influenced neonatal mortality. Considering infant mortality, the significantly adjusted determinants include maternal age, marital status, mothers’ education, place of residence, religion, child’s sex, birth interval, weight at birth, the skill of birth attendant, delivery by caesarean operation or not. Also, maternal age, marital status, mothers’ education, place of residence, the zone of residence, child’s sex, birth interval, weight at birth, delivery by caesarean operation or not were the significant risk factors of under-five mortality.

## Discussion

In this study, we determined the trends and drivers of neonatal, infant and under-five mortalities in Nigeria using data from three consecutive NDHS in 2003, 2008 and 2013. We found that there was a consistent reduction in NMR, IMR, and U5M for each of the surveys years. These reductions might be a reflection of the gains of the efforts put in place by member states of the United Nations in achieving the millennium development goals (MDGs) between 2000 and 2015.

In Nigeria, the successes recorded may be as a result of the various interventions of government in ensuring wider immunization coverage for all vaccine preventable diseases. In the year 2002, Nigeria endorsed the United Nations Special Session (UNGASS) goals on children of achieving by the year 2010 full immunization of children under one year of age at 90% coverage nationally with at least 80% coverage in every district or equivalent administrative unit [19]. A national policy on sustainable development with the sole aim of reducing infant and under-five mortality rates was formulated between 2003 and 2006 [17,20]. The WHO in the year 2006 and 2007 also endorsed the introduction of Haemophilus Influenza b (Hib) and Pneumococcal conjugate vaccines respectively into all national immunisation programs [21].

Moreover, the pentavalent vaccines that protect against Hib, hepatitis b, diphtheria, tetanus, and whooping cough were introduced by the Nigerian government to the immunization schedule in 2011 [21]. It was postulated that with the introduction of the pentavalent vaccine, about 400,000 cases of Hib would be prevented and about 27,000 lives saved annually [22]. Studies have shown that the use of vaccines to prevent the occurrence of diseases such measles, diphtheria, pertussis, Hib, and pneumococcus, has the potential to largely reduce disease incidence in children [23,24].

Though, there was a consistent reduction in NMR, IMR, and U5M for each of the surveys years, their prevalence was still high. The decline recorded between 2003 and 2013 for NMR and IMR was slower than that for U5M. Previous studies corroborate our position that increases in child survival is more pronounced within the first five years of life compared to the proportions of childhood deaths between birth and 28 completed days [15,25]. It could be explained that interventions from government and other relevant agencies targeted towards the post-neonatal age may account for lower U5M [26].

Our findings showed that maternal characteristics play a significant role in child mortality. The NMR, IMR, and U5M were consistently higher among children whose mothers were younger than among those with older parents across the survey years. Naivety in child nurturing, physical immaturity and pregnancy complications are plausible factors responsible for this trend among younger mothers [27,28]. The relationship between mother’s education and childhood mortality across the three age ranges as pointed in our study is well documented in previous studies [16,29,30]. Children of less-educated mothers have a higher probability of dying at all ages [31,32]. The basis for this consistent findings was that educated mothers may likely have improved income, better health education and make healthier choices for their health and that of their children [29,30].

Also, findings from this study show that the mortality rates considered were higher in rural areas than the urban areas, and also higher among children from poorer households. A similar trend has been reported in Bangladesh [26], Burkina Faso [26], and Rwanda [26]. Rural-urban differentials have previously been elucidated in relation to environmental factors (unavailability of improved water sources and inadequate basic sanitation facilities) and limited access to healthcare, social and economic services [30,33,34]. Household wealth is a significant predictor of U5M [29,35]. Poverty is a driver of unclean fuel use in many low-and-middle-income countries [36]. Half of all deaths linked to acute respiratory infections in the year 2014 resulted from children exposure to fumes from unclean fuels [37].

Our study revealed that NMR, IMR and U5 mortalities were highest among women whose prenatal care was provided by an unskilled attendant. Evidence abounds in the literature that antenatal care for pregnant women provided by skilled professionals is essential for ensuring optimal health outcomes for mother and child [7,15,38]. Furthermore, we found that the risk of dying among children across the three age groups surveyed in our study was lower among females than males. Previous studies have revealed that new-born boys have a lesser biological advantage in survival over new-born girls [39]. Biological [27,40,41] and genetic [39] factors may be the likely explanation for increased risk of male deaths. In addition, delay in the maturity of the lung in the first week of life among male children promote the higher occurrence of respiratory infections among them than females [42].

A wider interval between succeeding births and birth order present lower mortality risks in our study. This is in line with other studies [15,43] that show a significant association between short birth intervals and poor child and maternal health outcomes. Quick sequence of births may erode the reproductive and nutritional status of the mother thus, leading to maternal depletion disorder and other associated health infections [42]. Birth order had been noted in the literature as an essential determinant of childhood death [34,44,45]. A similar study have also shown that lower ranked birth order presents a lower risk of deaths among under-five children [42].

In addition, our study revealed that women who used biomass/unclean fuel, water from unimproved sources and unimproved toilet facilities had experienced higher neonatal, infant and under-five deaths than those using clean fuel, improved water sources and improved toilet facilities respectively. Previous studies have pointed out that children <5 years of age living in houses using solid fuels have a higher probability of dying than those in houses using cleaner fuels [44–46]. Wealthy households are more likely to use water from improved sources [42]. Diarrhea disease in children has been reported to result from sourcing water from unimproved sources and poor sanitation practices [47].

## Conclusion

Findings from our study showed a reduction in the proportions of NMR, IMR, and U5 mortalities in Nigeria, and that many of the drivers of NMR, IMR, and U5 mortalities had varying degrees and trends of change between 2003 and 2013. Maternal age, mothers’ education, place of residence, child’s sex, birth interval, weight at birth, the skill of birth attendant, delivery by caesarean operation or not, use of unclean fuel, unimproved water and unimproved toilet significantly influenced neonatal, infant and under-five mortalities. Though these factors have earlier been pointed out in literature, not much progress has been recorded in the reduction of the burden of childhood deaths. Multi-sectoral interventions and comprehensive health policies targeted towards the drivers of childhood mortality revealed in our study should be instituted to improve child survival.
